# How Does Journalism Aid the Development of the Medical Profession

**Published:** 1886-03

**Authors:** 


### HOW DOES JOURNALISM AID THE DEVELOPMENT OF THE MEDICAL PROFESSION.

The New York *Medical Journal* says that "it regards the great distinctive service of American medical journalism as shown mainly in its counteracting influence in removing the pedantry shown in the medical colleges, and encouraging the expression of original thought in young men." Surely, it were a great thing to remove the stupidity engendered by medical schools, and to draw forth the powers of original thought which are dormant in the young doctor. Of the truth of this view we have no question, nor can any editor of any experience or success fail to have many personal experiences in this sort of work. In the very best sense of that term, the medical editor is a teacher; and this, too, in causing others to work for the common good. The education follows from the efforts of the young doctor to learn something of profit or interest to the profession, and then place this before the profession in the most attractive shape. The medical editor, in order to make his journal a success, is compelled to get the best work expressed in the best way. Most of the older members of the profession have never learned to write, and as they become burdened with the cares of a large business, it becomes impossible for them to learn the art of writing. Much they possess of positive value to the profession, but from the defect of not being able to write with comfort, their knowledge dies with them. The medical editor can get little help from them. There are a considerable number of the members of the medical profession who could not write a decent article, if they had any distinct ideas to put in it. Obviously, the medical editor can do nothing with this class. But there is another class of doctors, who have the general culture and the brains, but are too modest to think of writing for the benefit of their seniors. From this class, the medical editor draws most of his working colaborers. By encouragement, by personal solicitation, by aid in matters of reference, by stimuli of ambition, of professional pride, by appealing to the sense of his obligations to do for the general profession that which lies in his power, many of this class are brought into active service in medical journalism. Having encouraged to habit of expression, the editor stimulates the habit of original research. Of course, different individuals will be stimulated in different directions. So, at last, the editor will have writers in every portion of the field of the art and science of medical surgery. Hence, it comes about that the editor sends men to work with the microscope, in the chemical laboratory, in the pharmaceutical laboratory, in the physiological laboratory, in the anatomical room, in the hospital, in the dispensary, in the tomes of medical literature of every language and of every age. In short, he has these men at work in every field congenial to them, and such that they can reach it.

In a very real sense, an editor is like a captain of a ship—he shows his abilities not so much in what he does himself, as in what he can get others to do. That there are not more really good medical journals, is due to the fact that there are really few medical men having the power of getting others to work in the fields leading to medical journalism.

When a young doctor has begun to realize that he can talk to the entire medical profession, life and study take an entirely new aspect. The day of small things is past, and the day of an enlarged and enlarging manhood has come to him. One who does realize this truth will never write a poor article for publication. The poor articles come from quite a different sort of men. These the medical editor gradually weeds out.

Of this direct and indirect influence upon the conduct of medical colleges, and upon medical societies and medical publishers, writers of medical books, and the relations of medical men, we have not time to speak. But in all these things the medical journal is the means by which the process of both good and bad education goes forward. Out of all these educational processes the medical profession is slowly rising higher in its development.

To every young man who would make the most of his powers, we say: think, observe, and write for the medical press constantly. It may be that one article a year is all that any particular person can produce. It may be that longer time will be required, but whether the time be long or short, be sure to begin and keep up the habit of correct thinking, constant study and correct and frequent writing.—*Detroit Lancet.*

